# Progress in Surgery

**Published:** 1901-10-19

**Authors:** 


### Progress in Surgery.

(*Continued from page* 34.)

**Gonorrhœal Myositis.**—Ware[4] has described a case of this rare and interesting complication of gonorrhœa. The patient, a man of 35, had contracted gonorrhœa two months previously. In the fourth week he developed fever and synovitis of the left knee, which got well in three weeks. He complained of severe pain about the shoulder. There was fulness there, and passive abduction caused spasm of the abductors. An induration the size of a walnut was felt in the muscles of the posterior axillary border, the skin over it being movable. Shreds containing gonococci were obtainable from the posterior urethra, but there were no subjective symptoms of gonorrhœa at this time. Micturition was frequent. The mass grew and became intensely painful. It was incised under the impression that pus was present. There was no pus, but turbid serum oozed out which was used for bacterial inoculations and cover-glass preparations. Also a piece of muscle was excised. The wound was left open and did not close for six weeks. Pain was relieved by the incision, but the induration extended and involved the whole of the muscle—*i.e.* the latissimus dorsi—to its insertion. When the wound closed the induration gradually disappeared. No cultures were obtained in the tubes, but the cover-glass preparations showed typical gonococci, though only sparsely. No other organisms were present. The muscle fragment showed interstitial myositis very beautifully. Sections of the muscle suitably stained showed numerous gonococci. Ware quotes three recorded cases of gonorrhœal myositis, the diagnosis having been based solely on clinical observation. It is considered probable that a large number of cases of myositis reported in females, classed as pyæmic following the puerperium, rest on a basis of gonorrhœal infection. Thus the sclerotic process so peculiar to gonorrhœa of the joints, urethra, and epididymis, again shows itself in the muscles. Batut is quoted as recording a case where myositis ossificans of the brachialis anticus occurred in a patient with gonorrhœal arthritis. The location of the gonococcus in these cases is explained either as a metastasis or as an extension of inflammation from adjoining joints or bones. The latter seems most likely, as in most cases the adjacent joints were affected. Ullman has shown that bones can be the seat of a gonorrhœal osteo-myelitis. The poison may travel from the bones or joints along the tendon lymphatics.

**Some Points in the Symptomatology of Syphilis.**—Thomas B. Futcher[5] has written on the association of fever with syphilis and describes three cases in which syphilis explained an otherwise obscure febrile attack. Syphilitic fever may occur very rarely three or four weeks before the secondary eruption. It usually occurs at the onset of the secondary symptoms, or about a week before, and it may occur at any time during the secondary or tertiary stages. The first case Futcher mentions was that of a woman who had four short febrile attacks, with a high temperature, between September 28 and October 14. The first was explainable as due to a pelvic abscess, but not the subsequent ones, and for these no other cause was discoverable except syphilis. The latter was diagnosed on the appearance of a rash on October 21. Thus the eruption did not appear until four weeks after the onset of the fever, and not until the temperature was normal. In the next case there was a temperature lasting a month which was reduced to normal by giving iodide of potash after clavicular swellings had appeared. The third case was of fever in the tertiary period also, the patient having had syphilis 28 years previously. The sternum and left shoulder joints became swollen, and iodide of potash brought the temperature down. M. G. Babinski and M. Charpentier[6] have recently related four cases which support their contention that permanent abolition of the pupillary reflexes, especially that of light —the Argyll-Robertson pupil—is a pathognomonic sign of acquired or hereditary syphilis. These writers think this sign shows that the central nervous system has been attacked by syphilis and that the patient is "a candidate for locomotor ataxy, general paralysis, or confirmed cerebro-spinal syphilis." Watch should therefore be kept upon the pupils in cases of suspected syphilis, and specific treatment be at once adopted if the sign be found. A. A. Scot Skirving[7] has noticed several cases of secondary syphilis in which itching of the fauces has been present. This was not a mere tickling, but a definite itchiness comparable to the ordinary feeling in the skin. In two cases, though there was some pain in the throat, especially with swallowing, it was the itchiness that was mostly complained of. One of the patients said he felt "inclined to tear the throat out." In these cases the itchiness was felt most over the tonsils, but also over the pillars of the fauces and posterior part of the side of the tongue. This itchiness affords an interesting contrast to the non-itchiness of the cutaneous syphilides.

**Primary Syphilis of the Conjunctiva.** — Carlo Fraginele[8] states that this condition is less rare than it is supposed to be. It is usually due to kissing, is most frequent at the inner canthus of the eye, and occurs mostly between 20 and 30 years of age. It is characterised by an ulceration of varying size associated with catarrhal conjunctivitis. Its diagnosis is said to be easy, though it might be mistaken for gumma or epithelioma.

**Syphilis in the Well-to-do.**—This is the title of a paper by J. A. McDonald (Conn.),[9] in which he states his conviction that the disease as it occurs in private practice is a mild affection and subjects its victims to less pain, mutilation, and loss of time than many a trivial complaint. To justify this statement he analyses 150 consecutive cases he has treated ; 65 per cent. had only mild lesions, including papular eruptions and mucous patches ; 26 per cent. showed some destructive lesions including pustular eruptions leaving scars, and necrosis of bones. Finally, in 6 per cent. the symptoms were severe for a time but finally cleared up completely leaving no trace. This class included those in which the hair fell out conspicuously, laryngitis was troublesome, and in which there were transitory internal troubles. Of the second group only four patients had temporarily to give up work. Only 6 per cent. of the cases were in any sense formidable. The writer then adduces another series of cases treated in hospital practice to show that in patients who are not well-to-do syphilis is a more serious disease. Amongst these patients he found 51 per cent. presenting destructive lesions and 10 other bad cases.

[4] Amer. Journ. Med. Scs., July, 1901. [5] New York Med. Journ. June, and Lancet (Abst.), July, 1901. [6] *Vide* Abst. Lancet, July, 1901. [7] Brit. Med. Journ.. May, 1901. [8] *Vide* Abst. Med. Rec., July, 1901. [9] Med. Rec., May, 1901.

